# Increased Physical Activity Decreases Prevalence of Periodontitis: The Korean National Health and Nutrition Examination Survey (KNHANES VI) (2013–2015)

**Published:** 2020-02

**Authors:** Eun-Jeong KIM, Gyeong-Soon HAN

**Affiliations:** 1. Department of Preventive and Social Dentistry, School of Dentistry, Seoul National University, Seoul, Korea; 2. Department of Dental Hygiene, College of Health Science, Gachon University, Incheon, Korea

## Dear Editor-in-Chief

Periodontitis is one of the most common chronic diseases worldwide. The risk factors for periodontitis are not only systemic diseases, but also health behaviors, such as smoking, stress, nutrition, and physical activity (1). Regular physical activity improves the quality of life and enhances general health and well-being (2). Increased physical activity is associated with improved insulin sensitivity and glucose metabolism (3), improve the quality of sleep, and to play a role in the management of anxiety and stress (4). In addition to all these health benefits, regular physical activity has a significant effect on reducing health care costs by reducing hospital visits and reducing medication needs (5). Studies have investigated the effect of regular physical activity on reducing the risk of some chronic diseases. However, only a limited number of studies have examined the relationship between physical activity and periodontitis.

Therefore, this study examined the potential relevance of Korean national representative samples by using the sixth Korean National Health and Nutrition Examination Survey (KNHANES) data. The aim of this study was to evaluate the association between physical activity and periodontitis after controlling for the socioeconomic status as well as general and oral health status and behaviors in a representative Korean adult population.

We examined 13,473 subjects who participated in the Korea Nation Health and Nutrition Examination Survey (2013–2015). Physical activity was defined according to the questionnaires. Trained dentists assessed the periodontal. A complex sampling design and multiple multivariate logistic regression analyses were sequentially applied to assess the association between periodontitis and physical activity. Stratified analyses were performed to identify specific risk groups.

Overall, 4,327 subjects were classified as having periodontitis. A crude analysis revealed that periodontitis was associated with physical activity. Compared to the participants without periodontitis, those with periodontitis were significantly older; were mostly males; bad perceived general health status, and inactive physical activity; and were never drinkers and smokers. Participants with periodontitis had a higher prevalence of hypertension, diabetes mellitus, and obesity. After adjusting for sociodemographic factors, the strength of this association remained significant in models 1 and 2 (Tables 1, 2).

**Table 1:** Univariate comparisons of the characteristics of subjects based on periodontal status

***Variable***	***Number***	***Periodontitis***				***OR (95% CI)***
		***Lower CPI***		***Higher CPI***		
		***N***	% (95% CI) ^*^	***N***	% (95% CI) ^*^	
Age (yr) ^†^						
20–39	3804	3402	89.5 (88.0–90.8)	402	10.5 (9.2–12.0)	1
40–59	5291	3406	62.7 (60.7–64.7)	1885	37.3 (35.3–39.3)	4.70 (4.03–5.48)
>60	4378	2338	52.6 (50.2–55.0)	2040	47.4 (45.0–49.8)	5.57 (4.60–6.74)
Sex^†^						
Male	5682	3447	65.5 (63.7–67.3)	2235	34.5 (32.7–36.3)	1.45 (1.28–1.64)
Female	7791	5699	75.7 (74.2–77.3)	2092	24.3 (22.7–25.8)	1
Hypertension^†^						
No	10384	7502	74.7 (73.2–76.0)	2882	25.3 (24.0–26.8)	1
Yes	3089	1644	51.8 (49.2–54.5)	1445	48.2 (45.5–50.8)	1.27 (1.12–1.43)
Diabetes mellitus^†^						
No	12311	8571	72.3 (70.8–73.7)	3740	27.7 (26.3–29.2)	1
Yes	1162	575	49.1 (45.6–52.6)	587	50.9 (47.4–54.4)	1.23 (1.05–1.44)
Obesity^†^						
Underweight	557	454	84.2 (80.4–87.3)	103	15.8 (12.7–19.6)	0.82 (0.62–1.09)
Normal	8477	5974	73.4 (71.8–74.8)	2503	26.6 (25.2–28.2)	1
Obese	4439	2718	63.8 (61.7–65.8)	1721	36.2 (34.2–38.3)	1.30 (1.18–1.43)
Perceived general health status^†^						
Good	4032	2940	76.1 (74.2–77.9)	1092	23.9 (22.1–25.8)	1
Ordinary	6842	4571	69.4 (67.7–71.1)	2271	30.6 (28.9–32.3)	1.15 (1.03–1.28)
Bad	2599	1635	64.6 (61.9–67.2)	964	35.4 (32.8–38.1)	1.08 (0.93–1.25)
Physical activity^†^						
Inactive	10112	6807	69.9 (68.3–71.4)	3305	30.1 (28.6–31.7)	1.22 (1.10–1.34)
Active	3361	2339	73.0 (70.8–75.1)	1022	27.0 (24.9–29.2)	1
Alcohol consumption^†^						
No	1731	1060	62.4 (59.2–65.5)	671	37.6 (34.5–40.8)	1
Yes	11742	8086	71.7 (70.2–73.1)	3656	28.3 (26.9–29.8)	0.83 (0.72–0.96)
Smoking status^†^						
Current	2483	1433	61.2 (58.6–63.7)	1050	38.8 (36.3–41.4)	2.22 (1.92–2.56)
Past	2689	1645	65.5 (63.1–67.7)	1044	34.5 (32.3–36.9)	1.27 (1.10–1.46)
Never	8301	6068	76.4 (74.8–77.9)	2233	23.6 (22.1–25.2)	1

CPI, Community Periodontal Index; OR, odds ratio

Values are presented as number (%).

* Weighted percent, 95% confidence interval (CI), and *p*-value obtained using the chi-square test.

Periodontitis: lower CPI, CPI lower than or equal to “code 2”; higher CPI, CPI greater than or equal to “code 3”.

Obesity: normal, body mass index (BMI)=18.5 to <25; underweight, BMI<18.5; and obesity, BMI ≥25.0 kg/m^2^.

Physical activity: inactive, the number of days per week strenuous activities are conducted and grouped as none; active: more than 1 day per week of strenuous activity.

† Statistical significance at *P*<0.05 by using the chi-square test

**Table 2:** Adjusted odds ratios (OR) and 95% confidence intervals (CI) between periodontitis and physical activity in multiple models

***Variable***	***Number***	***OR (95% CI)***		
		***Model 1^a^***	***Model 2^b^***	***Model 3^c^***
Physical activity				
Inactive	10,112	1.22 (1.10–1.34)	1.19 (1.06–1.33)	1.11 (0.99–1.24)
Active	3,361	1	1	1

The dependent variable was periodontitis (higher Community Periodontal Index).

a Model 1 was unadjusted.

b Model 2 was adjusted for sex, age.

c Model 3 was adjusted for all variables in model 2 and smoking status, alcohol consumption, hypertension, diabetes mellitus, obesity, and perceived general health status.

Bold values denote statistical significance at *P*<0.05

An association was found between physical activity and periodontitis after adjustment for potential confounders. A healthy lifestyle, including physical activity, can help maintain good general health as well as oral health. Considering the health benefits of physical activity, dental health professionals should encourage patients to perform physical activity to improve their general and oral health.
